# The role of alveolar epithelial junctions in early lung injury and COVID-19-induced fibrosis

**DOI:** 10.1007/s00418-026-02533-2

**Published:** 2026-09-11

**Authors:** Julián Wiegner, Michael Kasper, Mirko H. H. Schmidt, Kathrin Barth

**Affiliations:** Institute of Anatomy, Medical Faculty “Carl Gustav Carus”, Technical University of Dresden, Fiedlerstr. 42, 01307 Dresden, Germany

**Keywords:** p120 catenin, COVID-19, Caveolin-1, P2X7R, Pulmonary fibrosis

## Abstract

Pulmonary fibrosis is a chronic progressive disease, caused by numerous factors, including severe acute respiratory syndrome coronavirus 2 (SARS-CoV-2). Damage to the alveolar epithelial barrier (formed by junctions between alveolar epithelial cells I) has been described as a key mechanism of early fibrosis and therefore could be crucial for COVID-19-associated sequelae. The integrity of adherens junctions is regulated by p120 catenin and is crucial for barrier regulation in epithelial-mesenchymal-transition-driven diseases such as fibrogenesis and cancer. Abundance and expression of p120 catenin and intercellular contact regulating proteins (caveolin-1, P2X7R) were investigated retrospectively in COVID-19 lungs (acute, chronic) compared with healthy controls and interstitial lung disease of different etiologies (*n* = 6). p120 Catenin was further investigated in early injury models using profibrotic agents in murine lung and human alveolar epithelial cell culture. p120 Catenin abundance was higher in chronic COVID-19 compared with acute COVID-19, suggesting a role in fibrotic development. The interacting proteins caveolin-1 and P2X7R were simultaneously decreased in COVID-19, indicating a role in early pathophysiology. Early epithelial damage showed no changes in p120 catenin and its phospho-Y228 site in cell culture whereas p120 catenin was reduced in murine lung culture. Thus, alterations in and interactions between p120 catenin and caveolae associated proteins may represent a potential mechanism underlying COVID-19-induced pulmonary fibrosis.

**Supplementary Information:**

The online version contains supplementary material available at https://doi.org/10.1007/s00418-026-02533-2.

## Introduction

Pulmonary fibrosis (PF) is a chronic, progressive pathologic condition in different lung diseases. It is characterized by significant extracellular matrix deposition, leading to interstitial expansion and capillary weakening, ultimately resulting in ventilatory failure (Bagnato and Harari 2015; Crouch 1990; Katzenstein and Myers 1998; Selman et al. 2001; Thannickal et al. 2004; Visscher and Myers 2006). Multiple abiotic and biotic factors can cause PF, including the severe acute respiratory syndrome coronavirus 2 (SARS-CoV-2), the causative agent of the coronavirus disease 2019 (COVID-19) (Bösmüller et al. 2021; Flaifel et al. 2022; Gorbalenya et al. 2020; Nurchis et al. 2020). PF, one of the most concerning post-acute sequelae of COVID-19, typically manifests subsequent to acute lung injury. This is particularly evident after acute respiratory distress syndrome, which represents a severe, acute form of microvascular lung injury (with the histopathologic finding of diffuse alveolar damage [DAD]), characterized by an immediate exudative inflammatory phase and a proliferative phase associated with alveolar epithelial cell (AEC) hyperplasia (Flaifel et al. 2022; Matthay et al. 2019; Tojo et al. 2023). It has been observed that patients with COVID-19 are at high risk of developing acute respiratory distress syndrome in about 30% of all COVID-19 cases (Azagew et al. 2023) followed by fibrotic changes in 7% of all confirmed COVID-19 cases (Li et al. 2021a; Groff et al. 2021; Yao et al. 2021) as a result of abnormal tissue repair responses during chronic inflammatory and profibrotic conditions (Bösmüller et al. 2021; Flaifel et al. 2022; Tian et al. 2020; Xu et al. 2020).

Initial and persistent damage of the alveolar epithelial barrier leads to cell differentiation (epithelial–mesenchymal transition [EMT]) and an increased production of profibrotic factors, which seems to be pivotal for fibrogenesis (Bagnato and Harari 2015). In COVID-19 and non-COVID-19 pulmonary fibrosis, fibroblasts are activated and contribute to the formation of excess connective tissue and drive disease progression (Saha and Talwar 2024). The inflammatory and profibrotic signaling pathways involved in COVID-19 associated and non-COVID-19 associated pulmonary fibrosis are intricate and interact with one another (Kajdaniuk et al. 2013). In particular, transforming growth factor (TGF-β) signaling promotes fibroblast activation and extracellular matrix production, which ultimately leads to scar tissue formation in the lungs (Bagnato and Harari 2015; D’Agnillo et al. 2021; Broekelmann et al. 1991; Vaz de Paula et al. 2021). To gain insight into the underlying mechanisms of COVID-19 and PF in general, we concentrated our efforts on the damage to AECs.

The initial epithelial damage at onset of disease is characterized by a loss of intercellular connections, which play a pivotal role in maintaining the barrier function of the alveolar epithelium (Kasper and Haroske 1996; Brune et al. 2015; Koval 2013). The alveolar epithelium, mainly formed by AEC type I (AECI) (Crapo et al. 1982), features three main types of intercellular connections: tight junctions (TJ), adherens junctions (AJ), and desmosomes (Garcia et al. 2018). The apicoadherent contacts (consisting of TJ and AJ) serve to separate the apical and basolateral membranes from each other, thereby maintaining cell polarity and separation of the external environment from the subepithelial tissue (Shin et al. 2006).

AJ consist of the calcium-dependent transmembrane protein E-cadherin, which connects neighboring cells (Takeichi 1991; Stappert and Kemler 1999; Nagafuchi and Takeichi 1988). On its cytoplasmic domain, it interacts with proteins of the catenin family (p120 catenin, *β*-catenin, plakoglobin) (Ishiyama et al. 2010; Ozawa et al. 1989). The protein p120 catenin is an armadillo repeat domain containing protein, which interacts dynamically with E-cadherin and regulates the integrity of AJ as well as enzyme activity of ras homologous guanosine triphosphatase (Rho GTPases) (Reynolds et al. 1994; Reynolds and Roczniak-Ferguson 2004; Anastasiadis and Reynolds 2001), thereby influencing EMT (Kourtidis et al. 2013), a hallmark of fibrosis and tumor progression (Lamouille et al. 2014). Early epithelial and AJ damage is associated with activation of catenin-mediated signaling pathways, impaired barrier function, and increased inflammation, thus indicating a pivotal involvement in early fibrotic development (Post et al. 2018; Orsulic et al. 1999; Heuberger and Birchmeier 2010; Kourtidis et al. 2013).

AJ are strongly associated with caveolae, which are invaginated specialized rafts and contain proteins of the caveolin family and a specific lipid composition for signaling processes that regulate the pathogenesis of tumor progression and PF (Razani et al. 2002; Koleske et al. 1995; Gabazza et al. 2004). Caveolin-1 (cav-1) is the best studied member of this protein family and is predominantly expressed in AECI in the lung, interacting with several signaling pathways, including TGF-*β* (Lamaze et al. 2017; Del Galdo et al. 2008). Cav-1 has antifibrotic effects as it negatively regulates collagen (Tourkina et al. 2008), TJ and AJ proteins, thereby affecting catenin-mediated signaling pathways (Xu et al. 2016; Lu et al. 2003). In AECI, the extracellular adenosine triphosphate (ATP) gated cation channel P2X7R has a central function in inflammatory and fibrotic diseases (Gentile et al. 2015; Adinolfi et al. 2018). P2X7R abundance affects immune response and collagen expression (Riteau et al. 2010; Hofmann et al. 2015; Galam et al. 2016). In our previous research, we demonstrated a direct interaction of P2X7R with cav-1, which leads to a mutual interaction of these fibrotic pathways (Barth et al. 2007, 2008).

To investigate the involvement of the alveolar epithelium, cell–cell contacts (p120 catenin), and profibrotic pathways (cav-1, P2X7R) in pulmonary fibrogenesis, changes in retrospective COVID-19 impaired human lung samples were studied. Further investigations were performed on the role of p120 catenin and its phosphorylation status in profibrotic, early injury models of human alveolar epithelial cell line NCI-H441, and murine lung tissue culture, using TGF-*β* and bleomycin (BLM). This study aimed to investigate quantitative changes in p120 catenin and the caveolae-associated proteins in COVID-19 impaired human lung tissue and in vitro injury models, thus clarifying the involvement of these profibrotic pathways in early lung damage.

## Methods

For a detailed description of all experimental procedures, please refer to supplemental material. For validation of antibody specificity, please refer to technical specifications of the commercial manufacturer.

### Human tissue samples

Human lung tissue samples were provided by the Tumor and Normal Tissue Bank (TNTB) of the National Center for Tumor Diseases / University Cancer Center / University Hospital Carl Gustav Carus Dresden (NCT/UCC/UKD) at Institute of Pathology of the University Hospital Carl Gustav Carus Dresden, Technical University of Dresden and used in accordance with the ethics approval of the Technical University of Dresden (EK 59032007; 06 March 2007 and BO-EK-175052020; 22 March 2020). Specimens were primarily assigned to groups based on the histological features described above, using sections from formalin-fixed, paraffin-embedded (FFPE) samples (Bösmüller et al. 2021; Flaifel et al. 2022; Tian et al. 2020; Travis et al. 2013): acute COVID-19 (*n* = 6), chronic COVID-19 (*n* = 4), interstitial lung disease (ILD) of different origin (*n* = 6), and healthy control lungs (*n* = 6). Hematoxylin and eosin (HE)-stained samples in the groups “chronic COVID-19” and “ILD” show characteristic fibrotic changes (myofibroblast proliferation, excessive ECM production, AEC hyperplasia, immune cell infiltration). See Supplementary Material for detailed information on group definition.

### Animal tissue culture

Wildtype C57BL/6 mice (*n* = 4) were purchased from Charles River Laboratories. The mice were housed in the animal facility of the Medical Faculty Carl Gustav Carus, Technical University of Dresden with food and water ad libitum. The experimental procedures were approved by the state directorate Dresden (DD24-5131/365/19 and TVT17/2016; 20 December 2016).

### Cell culture

Human lung NCI-H441 cells (Salomon et al. 2014; Kielgast et al. 2016; Hermanns et al. 2004) were cultivated in RPMI-1640 medium (ATCC), supplemented with 10% fetal calf serum at 37 °C with 5% CO_2_. For experimental conditions, the culture medium was supplemented with insulin, transferrin, selenous acid (ITS) and dexamethasone (Hermanns et al. 2004; Neuhaus et al. 2012). Cells were treated with 100 mU/mL BLM (STADApharm GmbH) or 1 ng/mL TGF-*β* (R&D Systems). For immunostaining, NCI-H441 cells were seeded on poly-L-lysine coated cover slips (Weinhold 2010). After treatment, cells were fixed with an acetone-methanol solution and stored at −20 °C.

### Western blot (WB) analysis

Protein concentration of lysed NCI-H441 cells was determined using the PierceTM bicinchoninic acid (BCA) Protein Assay Kit (Pierce Biotechnology). 20 µg of sample protein was mixed with 4× LI-COR^®^ loading buffer, heat-denatured, separated in a 10% sodium dodecyl sulfate (SDS)–polyacrylamide gel and transferred onto an Immobilon^®^-FL polyvinylidene difluoride (PVDF) membrane (Merck Millipore).

The total protein was stained with the Revert™ 700 total protein stain kit and detected on the Odyssey^®^ Fc imager. After de-staining, the membrane was blocked with Intercept^®^ (TBS) blocking buffer, incubated with the primary (Table 1) and secondary antibody (IRDye^®^ 680 goat anti-mouse immunoglobulin G [IgG], infrared dye (IRDye)^®^ 800 goat anti-rabbit IgG). Semi-quantitative analysis was performed using Image Studio™ Lite (all from LI-COR Biosciences) and statistical comparisons were only done on the same blot.

**Table 1:** Primary antibodies used for western blot, immunohistochemistry and immunofluorescence

Antibody	Dilution used		Host	Type	Supplier
	WB	IHC/IF			
Anti-caveolin 1 (D46G3) Product no.: #3267	–	1:160	Rabbit	Monoclonal	Cell Signaling Technology (Danvers (MA), USA)
Anti-p120 catenin (6H11) Product no.: 33–9700	1:500	1:8000 (PCLS–IHC) 1:800 (PCLS–IF) 1:20 (human)	Mouse	Monoclonal	Invitrogen (Carlsbad (CA), USA)
Anti-p120 catenin phospho-Y228 Product no.: PA5-40,286	1:500	1:1.000 (PCLS–IHC) 1:100 (PCLS–IF)	Rabbit	Polyclonal	Invitrogen (Carlsbad (CA), USA)
Anti-p120 catenin phospho-Y228 Product no.: C358960	–	1:20 (human)	Rabbit	Polyclonal	LifeSpan BioSciences, Inc (Seattle (WA), USA)
Anti-P2X7R product no.: 177 003	–	1:40	Rabbit	Polyclonal	Synaptic Systems (Göttingen)

*PCLS* precision-cut lung slices, *IHC* immunohistochemistry, *IF* immunofluorescence

### mRNA quantification using real-time reverse transcription polymerase chain reaction (PCR) (real-time RT-PCR)

For mRNA extraction, human FFPE tissue sections were processed using the NucleoSpin^®^ total RNA FFPE XS Kit (Macherey–Nagel GmbH & Co. KG). Complementary DNA was synthesized using RevertAid H Minus 1st Strand Synthesis Kit (Thermo Fisher Scientific Inc.) and a Techne TC-512 Thermal Cycler (Keison Products). Quantitative PCR was performed using the SsoAdvanced™ Universal Inhibitor-Tolerant SYBR^®^ Green Supermix on a CFX96 Touch Real-Time PCR Detection System (both from Bio-Rad Laboratories GmbH) (primer sequences are listed in Table 2). Relative mRNA expression levels were calculated using the ∆∆Ct method with *β*2-microglobulin (*B2M*) and TATA box binding protein (*TBP*) as housekeeping genes.

**Table 2:** Primer sequences for RT-PCR

Gene	NCBI no.	Sequence	Length
huB2M	AF072097.1	5′-GGTTTCATCCATCCGACATTG-3′ 5′-GGTTCACACGGCAGGCATAC-3′	166 bp
huCAV1	BT007143.1	5′-AACCGCGACCCTAAACACCT-3′ 5′-CCTTCCAAATGCCGTCAAAA-3′	103 bp
huCTNND1	BC075795.1	5′-GCCTCTGCCCTGGATTGGTT-3′ 5′-ATGGAAGGGCGGGAGGAGAT-3′	144 bp
huP2RX7	BC007679.2	5′-AGTCACTCGGATCCAGAGCATGA-3′ 5′-CACTCACCAGAGCAAAGCAAACG-3′	92 bp
huTBP	BT019657.1	5′-CGGTTTGCTGCGGTAATCAT-3′ 5′-GGCTCCTGTGCACACCATTT-3′	87 bp

### Culture and paraffin embedding of precision-cut lung slices (PCLS)

PCLS were generated as described previously (Held et al. 1999; Ebeling et al. 2014; Hofmann et al. 2015) with minor modifications. Briefly, C57BL/6 J mice were euthanized, exsanguinated and the lungs were perfused with 0.8% agarose. After isolation, the lungs were embedded in 3% agarose and sectioned into 500 µm-thick PCLS. Following treatment with 300 mU/mL BLM in DMEM/F12 medium, PCLS were fixed with 4% formalin, followed by incubation in 0.1 M Sørensen buffer and dehydration through an ascending ethanol series (70%, 96%, and 100%), xylene and paraffin. Finally, the sections were embedded (see supplementary material for the detailed protocol).

### Immunohistochemistry (IHC) using immunoperoxidase and immunofluorescence (IF)

For immunohistochemical staining, 5 µm sections from paraffin-embedded human and murine PCLS were deparaffinized and rehydrated by incubation in xylene, a descending ethanol series, and water.

Immunoperoxidase staining of murine PCLS was performed as previously described (Ebeling et al. 2014) using the VECTASTAIN^®^ Elite^®^ ABC Kit (Vector Laboratories). Following antigen retrieval, sections were incubated with 0.3% hydrogen peroxide, blocked with 1.5% normal serum, and subsequently incubated with the primary (Table 1) and secondary antibody. The sections were then incubated with the VECTASTAIN ABC reagent and visualized using 3,3’-diaminobenzidine (DAB). Counter-staining was performed with Mayer’s hemalum. The slides were washed in tap water, dehydrated through an ascending ethanol series and xylene, followed by mounting with DePeX.

For IF experiments, murine PCLS were additionally stained with biotinylated lycopersicon esculentum (tomato) Lectin (1:1600), followed by secondary antibody incubation with Texas Red^®^ Avidin D (1:400, both from Vector Laboratories) (Barth et al. 2006). After final staining with 4′,6-diamidino-2-phenylindole (DAPI) nucleic acid stain (Invitrogen), the slides were mounted with Fluoromount-GTM (Life Technologies Corp.).

For IF staining of human NCI-H441 cells, coverslips were thawed and blocked with 1% bovine serum albumin (BSA). Human lung tissue sections were additionally bleached in 6% H_2_O_2_ for 10 min after re-hydrating and subsequently subjected to antigen retrieval in EDTA buffer. The subsequent staining steps were performed as described for murine PCLS.

IF-stained specimens were imaged using a Leica DM6 B fluorescence microscope (Leica Microsystems GmbH) and analysis was performed using (Fiji is Just) ImageJ (US National Institutes of Health). For comparison of signal intensities, the mean fluorescence intensity (MFI) was quantified according to the protocol described by Shihan et al. (2021). Details regarding the definition of the region of interest are provided in the Supplementary Material.

### Statistical analysis

For paired experimental approaches, the control value was normalized to 1. The normality of each data set was assessed using Shapiro–Wilk test. Normally distributed data were analyzed using a two-tailed paired Student’s *t*-test for comparison between two groups or one-way analysis of variance (ANOVA) for comparison among multiple groups, followed by Bonferroni’s post hoc hypothesis testing. Nongaussian distributed data were analyzed using the Wilcoxon signed-rank for comparisons between two groups or nonparametric ANOVA (Friedman test for paired groups or Kruskal-Wallis test for unpaired data), followed by Dunn’s post hoc hypothesis testing for multiple comparisons. Statistical analyses were performed using GraphPad Prism. Statistical significance was defined as *p ≤ 0.05 and **p ≤ 0.01.

## Results

### p120 Catenin increases in chronic COVID-19

Since p120 catenin regulates AJ integrity and fibrotic remodeling through the activity of Rho GTPases (Reynolds et al. 1994; Reynolds and Roczniak-Ferguson 2004; Anastasiadis and Reynolds 2001; Lamouille et al. 2014), its expression was investigated in human lung tissue.

**Fig. 1:**
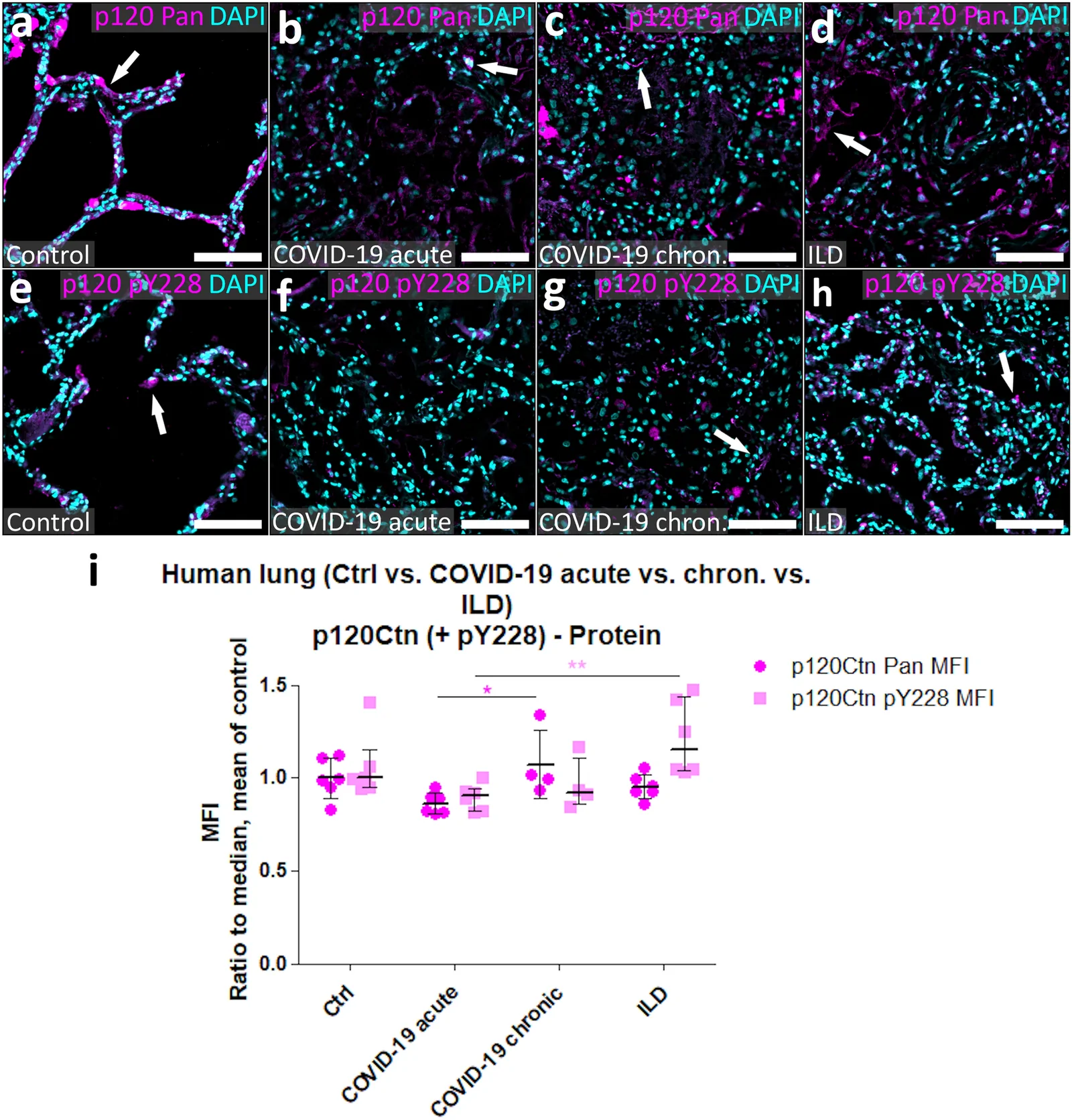
p120 Catenin protein abundance is increased in chronic COVID-19 compared with acute phase. (**a**–**h**) Human lung tissue sections from healthy control (**a**, **e**), acute COVID-19 (**b**, **f**), chronic COVID-19 (**c**, **g**), and interstitial lung disease (ILD, **d**, **h**) of different etiologies were stained for p120 catenin pan **a**–**d** and pY228 **e**–**h** (magenta), DAPI nucleic stain (cyan). Representative images are shown. Arrows indicate p120 catenin localization at the plasma membrane of AECs. (**i**) Quantitative analysis of the MFI of control (*n* = 6), acute COVID-19 (*n* = 6), chronic COVID-19 (*n* = 4) and ILD (*n* = 6), expressed relative to the control mean (pan: mean ± SD (*p* = 0.0354), pY228: median ± IQR (*p* = 0.0071)). *p < 0.05, **p < 0.01. Scale bars = 100 µm (**a**–**h**)

IF staining demonstrated that p120 catenin was localized predominantly at the plasma membrane of AECI and accumulated in intracellular aggregates of AECII (Fig. 1a-h). p120 Catenin pan protein abundance decreased during acute stage of COVID-19 compared with controls (86.3%) but increased significantly in chronic compared with acute stage (107.4% versus 86.3%, *p* = 0.0354). In contrast pY228 remained low (90.4% in acute, 92.3% in chronic COVID-19). No statistically significant difference in pan protein abundance was observed between COVID-19 acute and ILD group (95.3%). In contrast pY228 portion was significantly increased in the ILD group compared with acute COVID-19 (90.4% versus 115.1%, *p* = 0.0071, Fig. 1i).

The chronic COVID-19 group showed higher p120 catenin pan protein abundance compared with acute COVID-19.

### Cav-1 and P2X7R decrease in acute stage COVID-19

The AECI-associated proteins cav-1 and P2X7R play pivotal roles in PF (Lamaze et al. 2017; Del Galdo et al. 2008; Gentile et al. 2015; Barth and Kasper 2009; Burnstock 2006; Chen et al. 2004; Li et al. 2013) and were therefore investigated in human lung tissues from patients with COVID-19.

**Fig. 2:**
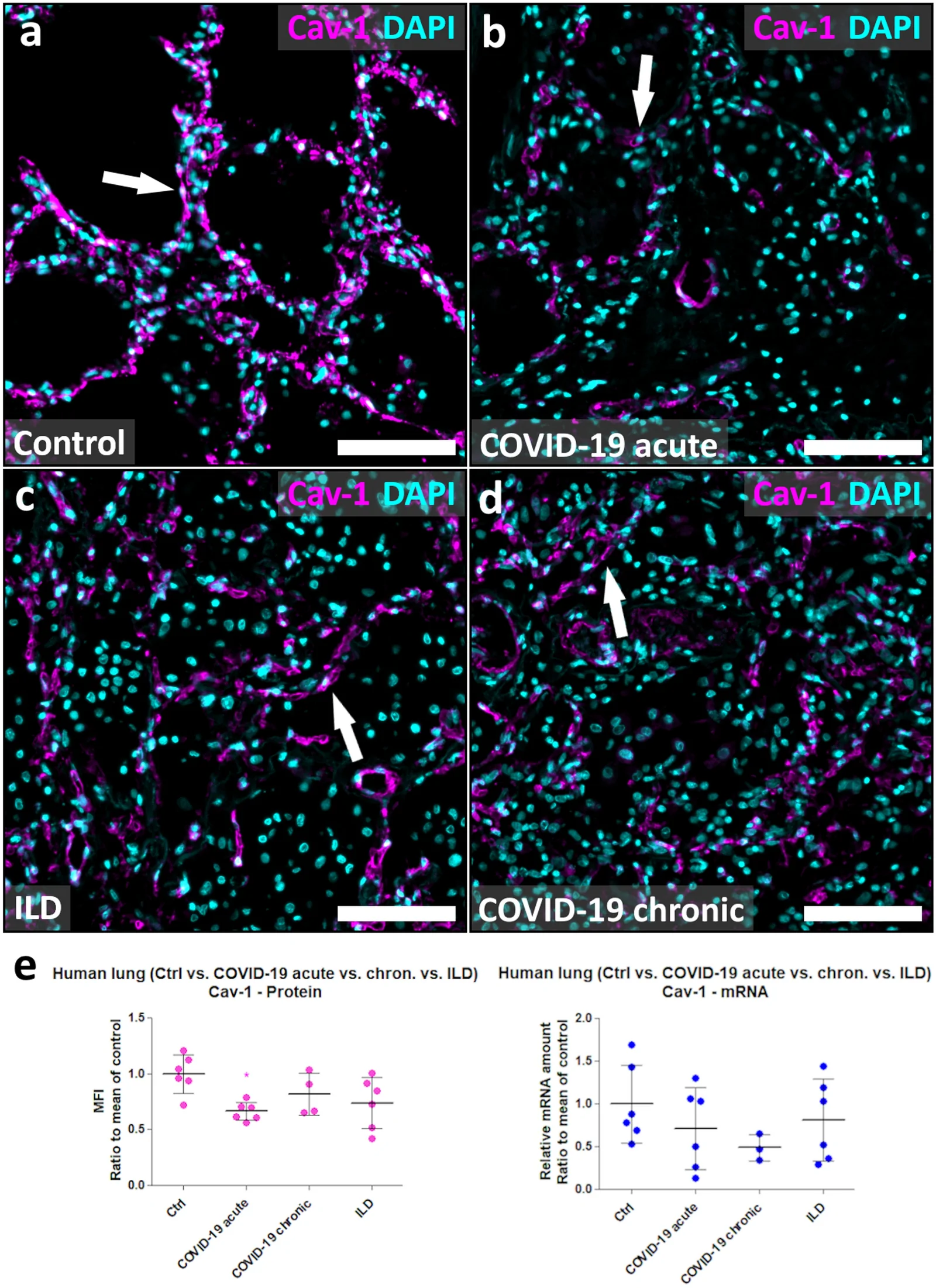
Cav-1 protein abundance and expression are decreased in acute COVID-19 compared with control. (**a**–**d**) Human lung tissue sections from healthy controls (**a**), acute COVID-19 (**b**), chronic COVID-19 **d** and interstitial lung disease (ILD, **c**) of different etiologies were stained for cav-1 (magenta), DAPI nucleic stain (cyan). Representative images are shown. Arrows indicate cav-1 localization at the plasma membrane of AECs. (**e**) Quantitative analysis of MFI and mRNA levels of control (*n* = 6; 6), acute COVID-19 (*n* = 6; 6), chronic COVID-19 (*n* = 4; 3) and ILD (*n* = 6; 6) expressed relative to the control mean (mean ± SD, MFI: *p* = 0.0226, mRNA: *p* = 0.4351). *p < 0.05. Scale bars = 100 µm (**a**–**d**)

IF staining demonstrated that cav-1 was localized predominantly at the cell membrane of AECI, with occasional intracellular aggregates (Fig. 2a-d). Cav-1 protein abundance was significantly reduced in acute COVID-19 to 66.5% of the control level (*p* = 0.0226). A similar trend was observed at the mRNA level (71.4%, *p* = 0.4351). In chronic COVID-19, the reduction compared with controls was less pronounced (81.6%), whereas mRNA expression decreased further (48.8%). In ILD of different etiologies, the protein abundance (74%) and mRNA expression (80.8%) of cav-1 were substantially reduced compared with controls (Fig. 2e).

**Fig. 3:**
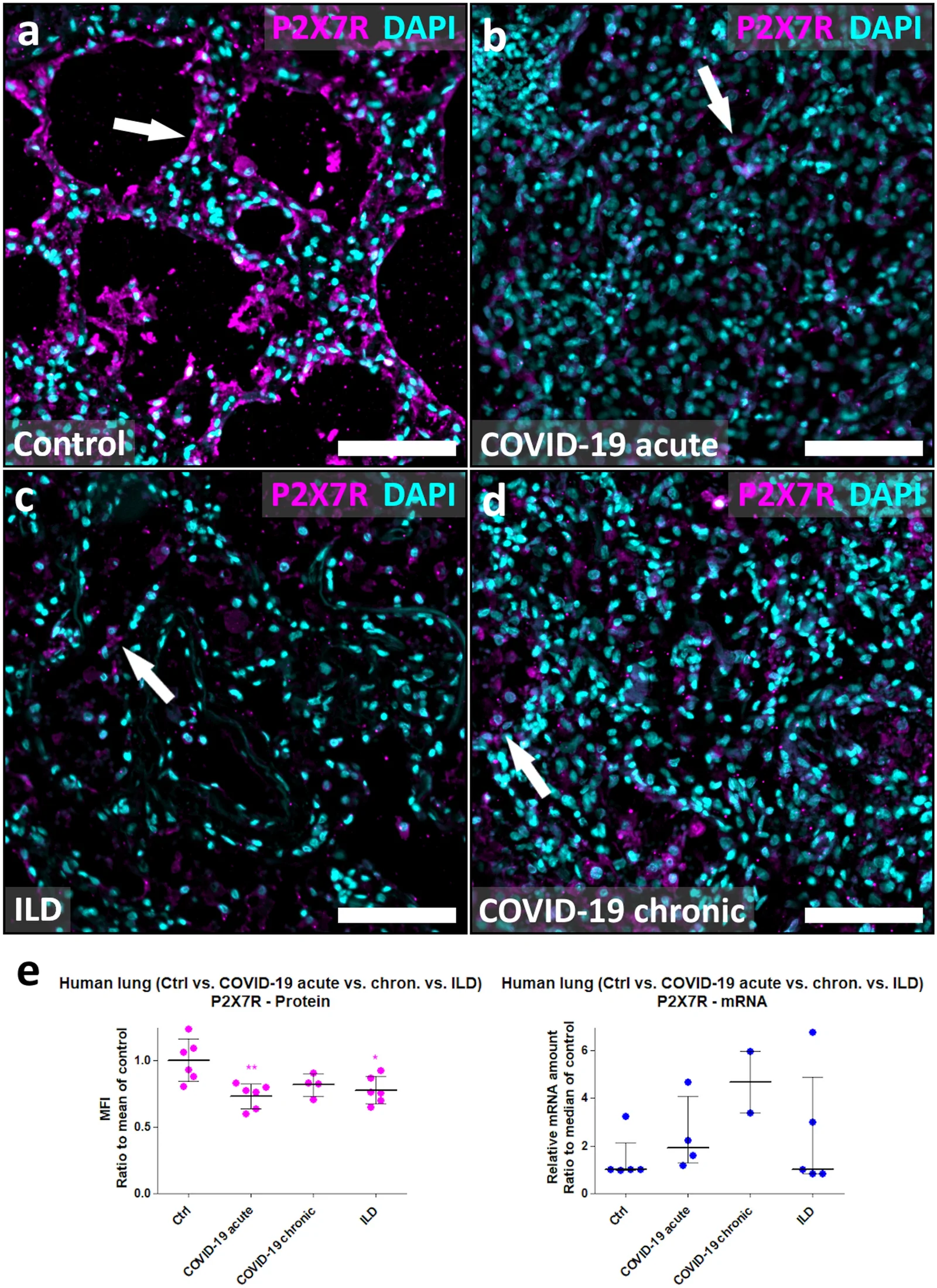
P2X7R protein abundance is decreased in acute COVID-19, but not in chronic stage, wherein expression increases. (**a**–**d**) Human lung tissue sections from healthy controls (**a**), acute COVID-19 (**b**), chronic COVID-19 (**d**), and interstitial lung disease (ILD, **c**) of different etiologies were stained for P2X7R (magenta), DAPI nucleic stain (cyan). Representative images are shown. Arrows indicate P2X7R localization at the plasma membrane of AECs. (**e**) Quantitative analysis of MFI, expressed relative to the control mean (mean ± SD, *p* = 0.0044) and mRNA levels expressed relative to the control median (median ± IQR, *p* = 0.1999); control group (*n* = 6; 5), acute COVID-19 (*n* = 6; 4), chronic COVID-19 (*n* = 4; 2), and ILD (*n* = 6; 5). *p < 0.05, **p < 0.01. Scale bars = 100 µm (**a**–**d**)

P2X7R was localized predominantly at the plasma membrane of AECI (Fig. 3a-d). P2X7R protein abundance was significantly reduced in acute COVID-19 (73.2%, *p* = 0.0044) and in ILD (77.5%) compared with controls, whereas only a modest reduction was observed in chronic COVID-19 (81.4%). In contrast, P2X7R mRNA expression was increased in COVID-19 (191% in acute stage versus 467% in chronic stage, *p* = 0.1999, Fig. 3e).

Overall, both P2X7R and cav-1 protein abundance were reduced during acute phase of COVID-19.

### p120 Catenin pY228 fraction increases in a time-dependent manner in BLM- and TGF-*β*-treated NCI-H441 cells

To investigate the effects of profibrotic factors on p120 catenin in early injury models of the alveolar epithelium, human NCI-H441 cells which represent a monolayer model of alveolar epithelial cells, were treated with BLM and TGF-*β*.

**Fig. 4:**
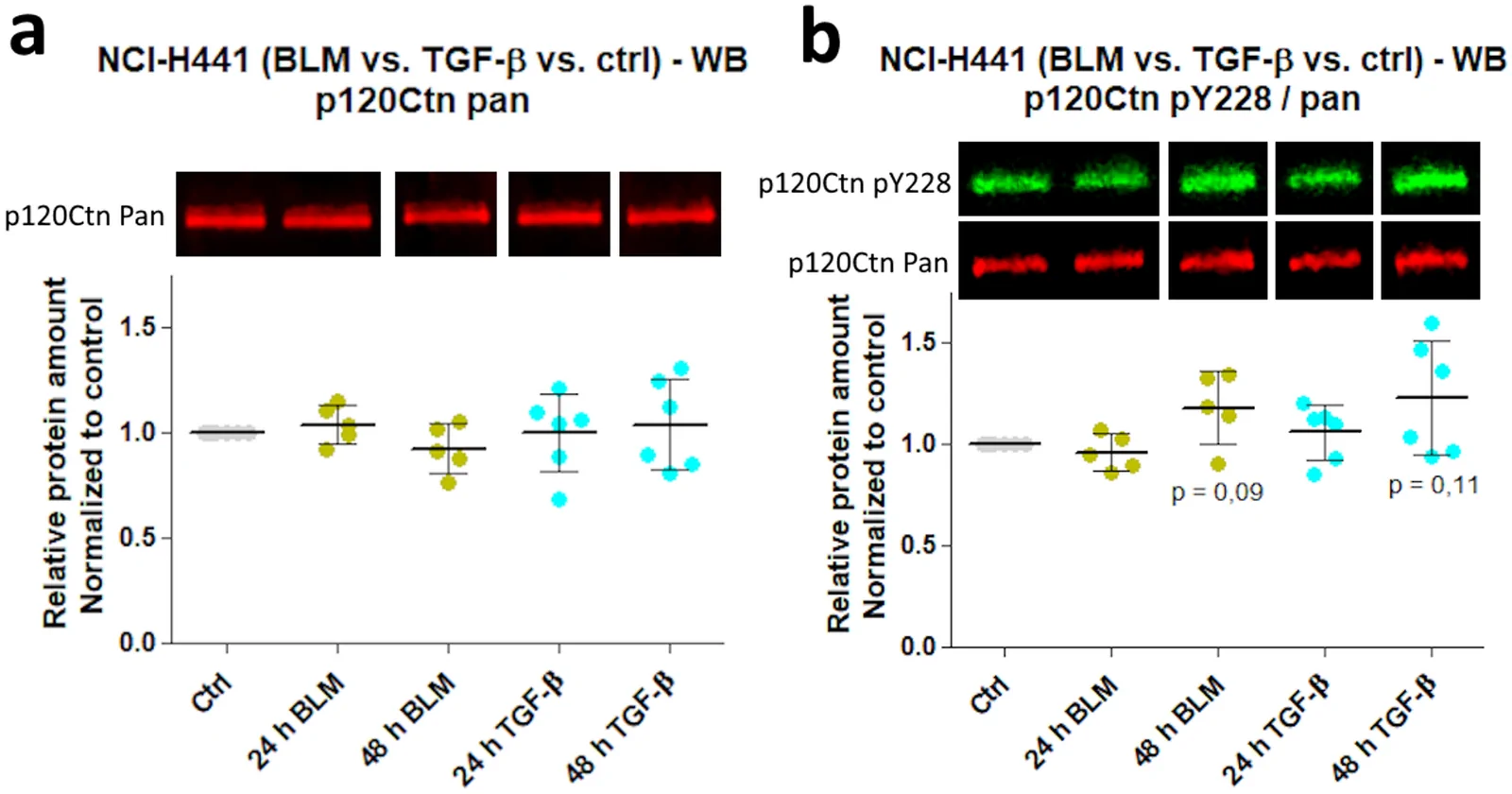
p120 Catenin pY228 fraction increases in a time-dependent manner in BLM- and TGF-*β*-treated NCI-H441 cells. (**a**–**b**) p120 Catenin pan and pY228 protein in NCI-H441 cells treated with BLM or TGF-*β* compared with control cells. Representative blots are shown. Band intensity was normalized to total protein staining of each lane (**a**) or to p120 catenin pan protein (**b**). Quantitative analysis of normalized protein amount of control and BLM- (*n* = 5, pan 24 h: *p* = 0.4134, 48 h: *p* = 0.2097, pY228 24 h: *p* = 0.36, 48 h: *p* = 0.0893) and TGF-β-treated (*n* = 6, pan 24 h: *p* = 0.9531, 48 h: *p* = 0.6859, pY228 24 h: *p* = 0.3565, 48 h: *p* = 0.1068) NCI-H441 cells is expressed relative to control mean (mean ± SD)

Only minor, nonsignificant changes in p120 catenin pan protein were observed following treatment with BLM or TGF-*β*. In comparison with the control, p120 catenin pan protein abundance was 103.7% after 24 h of BLM treatment (*p* = 0.4134) and 92.2% after 48 h (*p* = 0.2097), while TGF-β treatment led to 99.5% of p120 catenin pan protein abundance after 24 h (*p* = 0.9531) and 103.8% after 48 h (*p* = 0.6859), (Fig. 4a). In contrast, the pY228 fraction (normalized to p120 catenin pan protein) showed a time-dependent trend toward increased phosphorylation in both BLM- (96% in 24 h (*p* = 0.36) versus 117.7% in 48 h (*p* = 0.0893)) and TGF-*β*-treated (105.6% in 24 h (*p* = 0.3565) versus 122.5% in 48 h (*p* = 0.1068)) NCI-H441 cells (Fig. 4b).

**Fig. 5:**
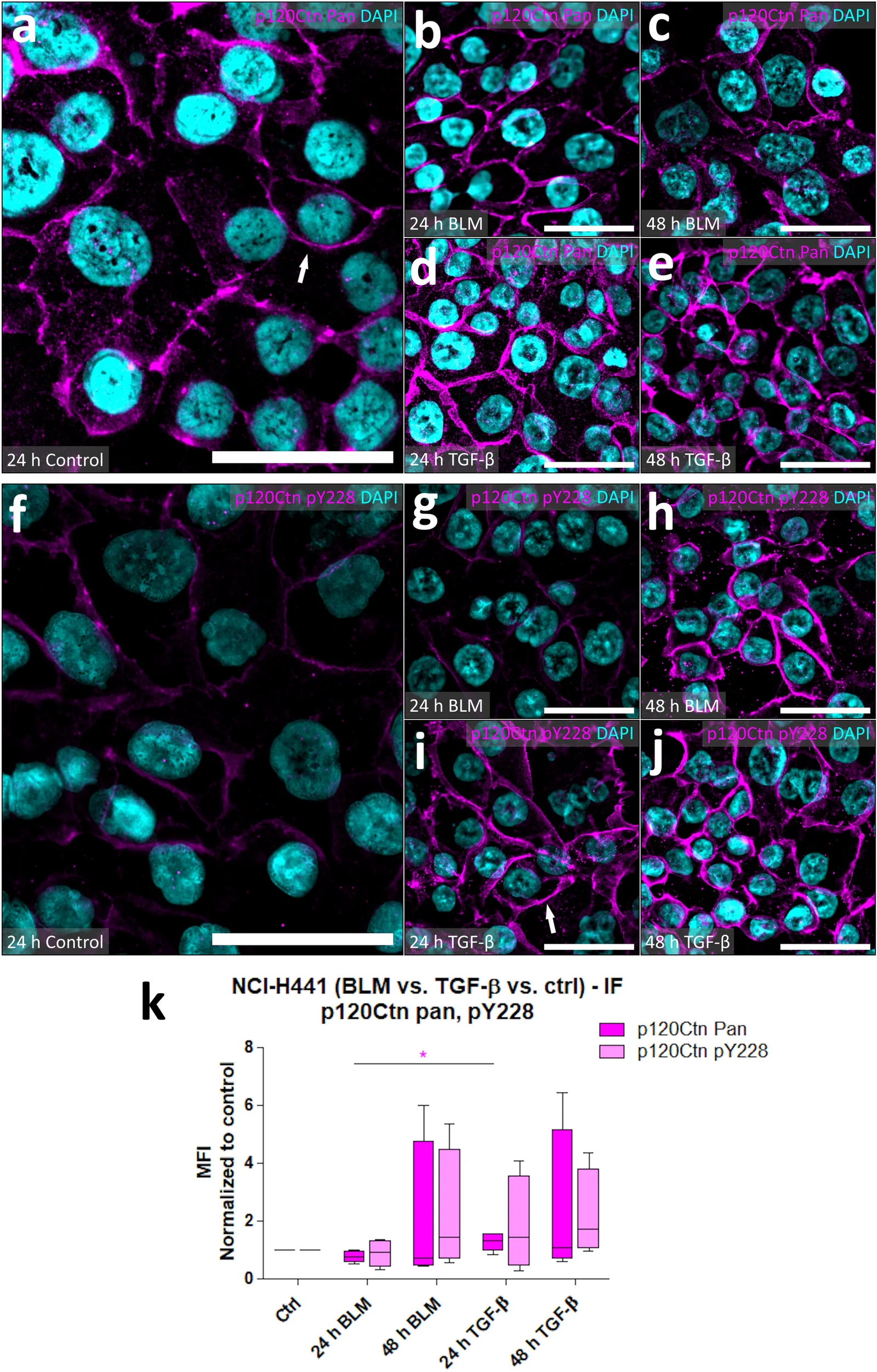
p120 Catenin pan protein and pY228 abundance remain unchanged at the plasma membrane of BLM- and TGF-*β*-treated NCI-H441 cells. (**a**–**j**) NCI-H441 cells, treated with BLM or TGF-*β*, compared with control cells, were stained for p120 catenin pan (**a**–**e**) and pY228 (**f**–**j**) (magenta), DAPI nucleic stain (cyan). Representative images are shown. Arrows indicate p120 catenin localization at the plasma membrane. (**k**) Quantitative analysis of MFI of control and BLM- (pan 24 h: *p* = 0.1067, 48 h: *p* = 0.875, pY228 24 h: *p* = 0.6834, 48 h: *p* = 0.3458) and TGF-*β*-treated (pan 24 h: *p* = 0.1795, 48 h: *p* = 0.413, pY228 24 h: *p* = 0.3994, 48 h: *p* = 0.2183) NCI-H441 cells (*n* = 4, pan 24 h BLM versus TGF-β: *p* = 0.0276), expressed relative to the control median (box-and-whisker-plot). *p < 0.05. Scale bars = 50 µm (**a**–**j**)

To further determine whether the membrane-associated pool of p120 catenin was altered, fixed NCI-H441 cells were immunostained for p120 catenin as well as lycopersicon esculentum (tomato) lectin. IF staining demonstrated that p120 catenin localized predominantly at the plasma membrane with only minor cytoplasmic aggregates (Fig. 5a-j).

MFI was quantified within a defined region of interest corresponding to the lectin-positive area, excluding the DAPI-positive area and thereby restricting the analysis to the plasma membrane. p120 Catenin pan protein abundance at the plasma membrane did not change significantly following treatment with BLM after 24 h (76.9%, *p* = 0.1067) and 48 h (73.3%, *p* = 0.875) or TGF-*β* after 24 h (133.9%, *p* = 0.1795) and 48 h (109.9%, *p* = 0.413), although the data showed considerable variability. However, NCI-H441 cells treated with TGF-β for 24 h exhibited significantly higher membrane-associated p120 catenin abundance than BLM-treated cells (133.9% versus 76.9%, *p* = 0.0276). Compared with the control condition, p120-catenin pY228 abundance was 91.9% after 24 h of BLM treatment (*p*=0.6834) and increased to 146.1% after 48 h (*p*=0.3458). Similarly, following TGF-*β *treatment, p120-catenin pY228 abundance reached 144.3% of control levels after 24 h (*p*=0.3994) and 172.2% after 48 h (*p*=0.2183; Fig. 5k).

Overall, p120 pan abundance remained largely unchanged in both whole cell lysates and at the plasma membrane. In contrast, WB analysis indicated a modest increase in the pY228 fraction, although this increase was not reflected in the membrane-associated protein.

### p120 catenin decreases in BLM-treated murine PCLS

To investigate the effects of a profibrotic environment on p120 catenin in a three-dimensional (3D) model of early alveolar injury in the absence of systemic humoral influences, murine PCLS were treated with BLM.

**Fig. 6:**
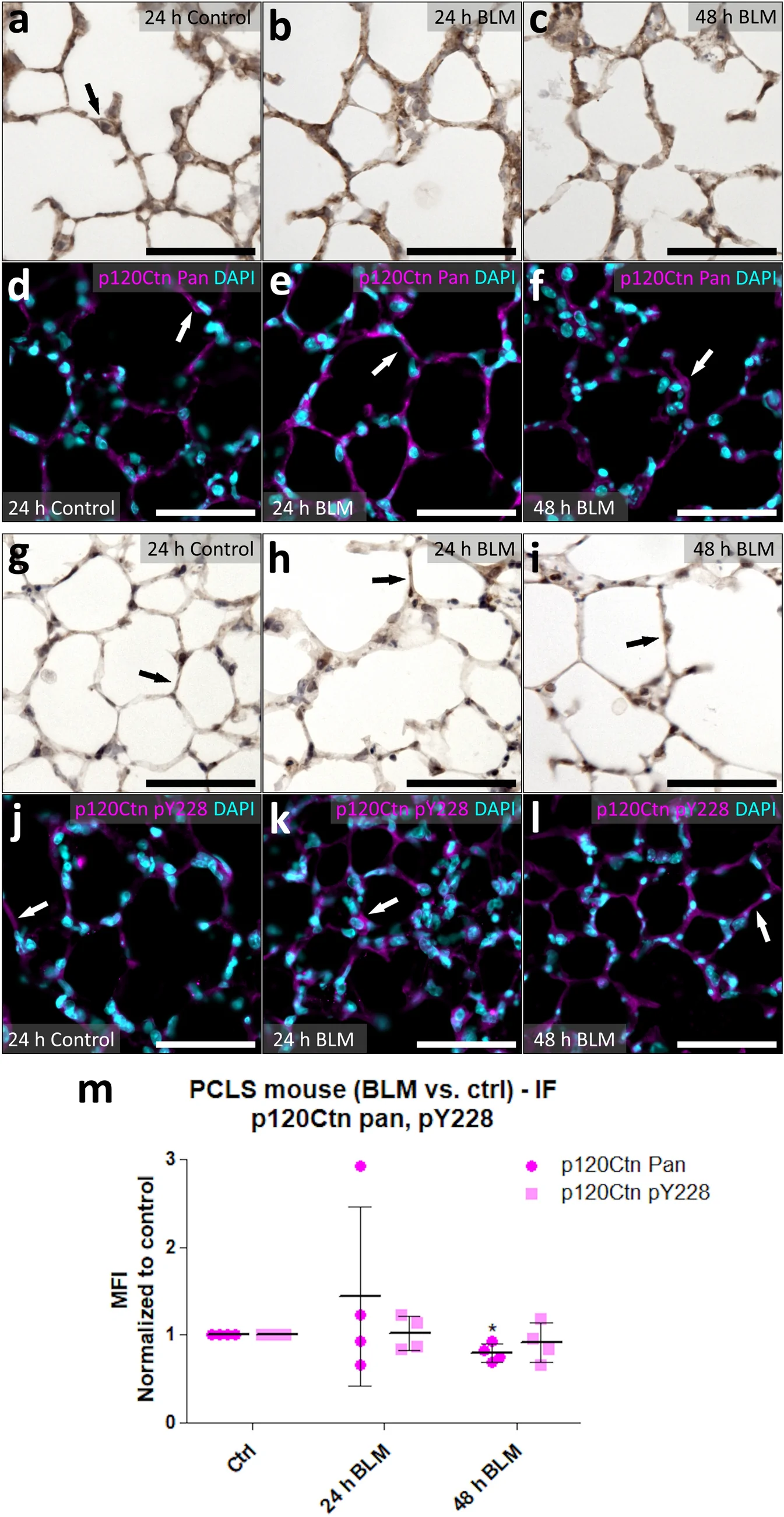
p120 catenin protein abundance decreases after 48 h of BLM treatment in murine PCLS compared with untreated controls. A-l PCLS, treated with BLM compared with untreated control slices, were stained using immunoperoxidase (**a**–**c**), (**g**–**i**), and IF (**d**–**f**), (**j**–**l**), p120 catenin pan (**a**–**f**) and pY228 (**g**–**l**) (magenta), DAPI nucleic stain (cyan). Representative images are shown. Arrows indicate p120 catenin localization in AECs. (**m**) Quantitative analysis of MFI of control and BLM-treated murine PCLS (*n* = 4), expressed relative to the control mean (mean ± SD, pan 24 h: *p* = 0.4507, 48 h: *p* = 0.0291, pY228 24 h: *p* = 0.8221, 48 h: *p* = 0.4957). *p < 0.05. Scale bars = 50 µm **a**–**l**

IHC demonstrated that p120 catenin was localized at the plasma membrane of both AECI and AECII with additional cytoplasmic staining in AECII (Fig. 6a-l).

p120 Catenin pan abundance increased after 24 h of BLM treatment (144%, *p* = 0.4507) but decreased significantly after 48 h compared with untreated controls (80.1%, *p* = 0.0291). No changes in p120 catenin pY228 abundance were detected after 24 h or 48 h (Fig. 6m, 102.4% in 24 h (*p* = 0.8221) and 91.4% in 48 h (*p* = 0.4957)).

These findings indicate that, in murine lung tissue cultured in absence of systemic humoral factors, p120 pan protein is reduced following prolonged BLM treatment.

## Discussion

PF is a chronic, multifactorial pathological condition in ILD and ultimately leads to progressive respiratory failure. Nevertheless, the underlying pathogenic mechanisms are not fully understood yet. Histological similarities between COVID-19 lungs and interstitial lung diseases of other origins, including myofibroblast proliferation, extracellular matrix production, and AECII hyperplasia (Bagnato and Harari 2015; Flaifel et al. 2022; Rendeiro et al. 2021; Valdebenito et al. 2021; Martinez et al. 2017) driven by profibrotic pathways (Vaz de Paula et al. 2021; Willis and Borok 2007; Ackermann et al. 2020) suggest common pathogenetic mechanisms.

Early and persistent damage to the alveolar epithelial barrier, formed by AJ seems to be crucial for the pathogenesis of PF (Tojo et al. 2023; Martinez et al. 2017; Takeichi 2014). Maintenance of physiological p120 catenin expression is essential for AJ integrity by interaction with E-cadherin (Reynolds et al. 1994; Kourtidis et al. 2013; Davis et al. 2003; Ireton et al. 2002). Several phosphorylation sites of p120 regulate its binding capacity to E-cadherin and the dissociation into the cytoplasm inducing downstream signaling pathways (Reynolds and Roczniak-Ferguson 2004).

p120 Catenin showed a significantly higher protein abundance in chronic COVID-19 compared with acute disease, suggesting that p120 catenin upregulation may contribute to fibrogenesis in COVID-19. It has previously been shown that p120 gets upregulated in profibrotic stages using TGF-*β* or BLM via transcriptional activity of SMAD and NF-κB pathways, inhibition of p120 expression attenuated fibroblast differentiation and pulmonary fibrosis (Zhang et al. 2019).

The phosphorylated form p120 catenin pY228 was more abundant in the ILD group compared with acute course of COVID-19. Phosphorylation of p120 at Y228 is known to decrease the binding capacity to E-cadherin while increasing its binding to RhoA and also affecting intracellular localization (Ding et al. 2019; Castaño et al. 2007). Moreover, increasing abundance of p120 catenin pY228 is associated with altered tumor behavior (Ding et al. 2019; Huveldt et al. 2013). Activity of Rho GTPases is crucial for EMT, as RhoA induces the formation of actin stress fibers while Rac1 and Cdc42 promote the formation of membrane protrusions, which together lead to dissociation of epithelial cell contacts and increased migration (Kuroda et al. 1998; Bhowmick et al. 2001). Nevertheless, a contribution of this specific phosphorylation to PF cannot be excluded.

To further investigate p120 catenin and the pY228 form, NCI-H441 cells were treated with TGF-*β*, the principal profibrotic cytokine driving PF (Willis and Borok 2007), and BLM, which is an antineoplastic drug. In addition, BLM is also an established model for lung injury in early stages of fibrogenesis due to its common side effect of lung toxicity, possibly leading to PF (Watson et al. 2018; Della Latta et al. 2015). Protein expression of p120 catenin pan protein was not affected in whole cell lysates of NCI-H441 treated with BLM or TGF-*β* for 24 h or 48 h, however, the pY228 fraction showed a small time-dependent increase. Quantification of membrane-associated protein abundance using MFI showed a time-dependent trend toward increased abundance of pan protein and pY228 fraction. Overall, despite a tendency to a higher pY228 fraction, no significant changes in p120 catenin and its pY228 fraction within 48 h were observed.

BLM-treated PCLS of WT murine lung showed a significant decrease of p120 catenin pan protein after 48 h. Binding of p120 catenin to E-Cadherin stabilizes AJ in the cell membrane, therefore loss of p120 catenin can also result in EMT (Kourtidis et al. 2013; Davis and Reynolds 2006), which is a common hallmark of PF (Lamouille et al. 2014) including COVID-19 (Rendeiro et al. 2021). Loss of p120 catenin exposes the E-Cadherin binding site for adapter protein 2 (AP-2) and the E3 ligase Hakai, thereby leading to increased endocytosis or ubiquitination of E-cadherin and impaired cell adhesion (Kourtidis et al. 2013). Previous investigations showed a reduction in p120 catenin abundance after short-term mechanical stress in murine AECs (Dai et al. 2013). Reduction of p120 catenin during short-term injury with BLM could be a possible mechanism leading to this profibrotic state. Our results therefore indicate a more complex regulation and role of p120 catenin and its phosphorylation pY228.

The AECI-specific protein Cav-1 is known to control catenin levels in injury models via activation of transcriptional pathways, thereby regulating AJ and alveolar barrier integrity (Ye et al. 2016). The human lung samples of acute COVID-19 showed a decrease in cav-1 protein abundance compared with the control group with similar proportional change in mRNA expression remaining reduced in chronic course of COVID-19 and ILD group. Previous investigations have shown a decrease of cav-1 based on the stained area in human COVID-19 lung (Vaz de Paula et al. 2021). Importantly, DAD occurring in COVID-19 leads to damage of AECs, which results in reduced abundance of epithelial markers in tissue slices (D’Agnillo et al. 2021). Because the region of interest was restricted to cav-1-positive epithelial cells, the reduced cav-1 signal in this compartment indicates that cav-1 is downregulated within the cells that express it. Cav-1 is known to decrease in early lung injury models (Menzel et al. 2022; Koslowski et al. 2004) and also in established PF (Del Galdo et al. 2008; Kasper et al. 1998; Shetty and Idell 2023). Cav-1 knockout mice show several profibrotic changes in the lung with increased fibrillar deposition and AECII hypertrophy as a potential result of altered regulation of gene transcription and cell differentiation (Drab et al. 2001). Knockdown of cav-1 has been shown to downregulate E-Cadherin and AJ integrity, therefore impairing epithelial cell adhesion, facilitating cell migration, and potentially increasing cytosolic abundance and above mentioned downstream signaling of p120 catenin or *β-*catenin (Song et al. 2012; Galbiati et al. 2000). In a spinal cord injury model, the protective effect of upregulating AJ proteins (including p120 catenin) is mediated by abundance of cav-1 (Ye et al. 2016). Thus, reduced cav-1 expression may represent an early event in alveolar injury leading to fibrogenesis in COVID-19. Since the endosomal cell entry of SARS-CoV-2 is dependent on cholesterol-rich lipid rafts which include caveolae, a potential colocalization, and therefore interaction between cav-1 and SARS-CoV-2 is possible (Li et al. 2021b).

P2X7R is an ATP-gated cation channel, which is involved in several pathophysiological processes, i.e., inflammation, neurodegenerative diseases, neoplasia, and fibrosis (Burnstock 2006; Riteau et al. 2010). Extracellular ATP acts as a damage associated molecular pattern (DAMP) through activation and short-term upregulation of P2X7R, thereby inducing further proinflammatory pathways (Gentile et al. 2015; Bläsche et al. 2012).

P2X7R staining showed reduced protein abundance in the COVID-19 and ILD group. Previous studies tied a cav-1 knockdown and knockout to a downregulation of P2X7R in pulmonary tissue. In turn, P2X7R abundance regulates cav-1. Additionally, a direct interaction between both proteins mediates the recruitment of P2X7R into AEC caveolae (Barth et al. 2007, 2008; Hofmann et al. 2015; Barth and Kasper 2009; Weinhold et al. 2010). The interaction between P2X7R and cav-1 may explain the decrement of P2X7R in COVID-19.

Our investigations have several limitations. The retrospective study design of the human specimen may introduce bias and confounding factors. The small sample size can lead to decreased statistical power. The interpretation of protein and mRNA changes in the human samples is limited by lacking information about pre-mortem treatment schemes (e.g., mechanical ventilation, oxygen therapy, pharmacologic interventions), which can impact protein and mRNA expression. The data were not included because the study focused on correlation between histological characteristics and protein changes. Postmortem mRNA degradation may influence relative mRNA levels, but this effect is likely minimized due to nonsignificant differences between the groups. The early lung injury models used in this study, including a single cell line (NC-H441), 3D in vitro model (murine PCLS), and postmortem lung specimens from patients with COVID-19, represent distinct experimental systems and different aspects of disease pathogenesis. Consequently, direct comparisons between these models should be made with caution. Since NCI-H441 cell culture lacks several interacting pulmonary cell types including myofibroblasts and endothelial cells, generalizability of the findings to human or murine lung tissue is also limited but facilitates the investigation of direct effects on alveolar epithelial cell. Nevertheless, common pathogenic processes have been described above. Additionally, fluorescence signal variability can lead to more statistical scattering. Experimental conditions were kept rigorously equal to minimize this effect.

## Conclusions

**Fig. 7:**
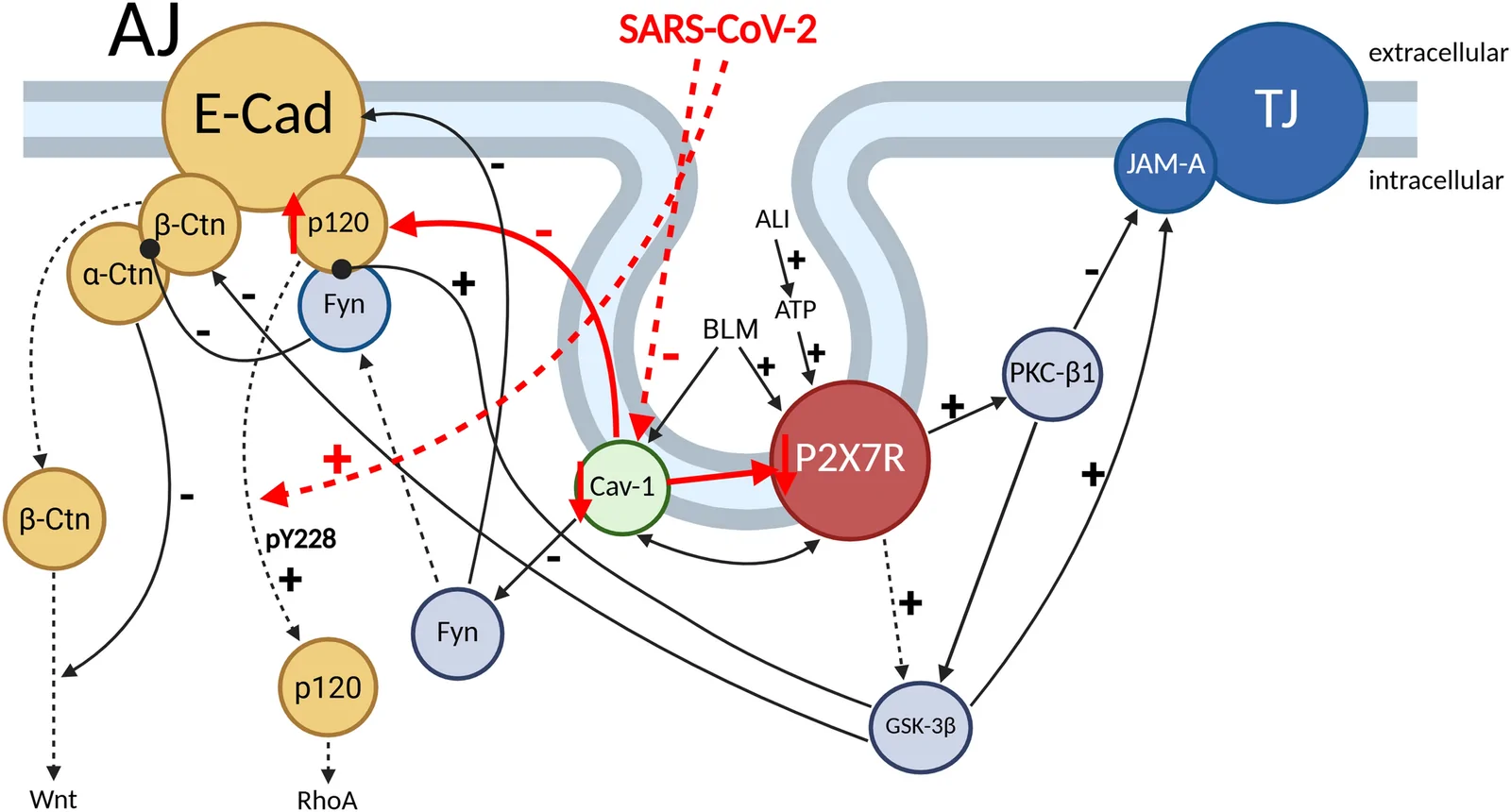
Potential interaction model between caveolae-associated proteins and intercellular connections. The caveolae-associated proteins cav-1 and P2X7R interact with each other and influence AJ and TJ. Acute lung injury induces ATP release, which activates P2X7R. Phosphorylations of *β*-Catenin or p120 catenin promote their dissociation from AJ and activate downstream signaling pathways, including Wnt and Rho GTPase signaling. Created in BioRender. Wiegner, J. (2026) https://BioRender.com/i61q937

Based on the findings of the present study, previous work from our group, and the available literature on the interaction between intercellular connections and caveolae-associated proteins we propose a model shown in Fig. 7. The caveolae-associated proteins cav-1 and P2X7R interact with each other and influence AJ and TJ proteins either by associating with them directly or through signalling partners such as downstream kinases (PKC-*β*1, Fyn, GSK-3*β*) (Barth et al. 2016; Wesslau et al. 2019; Lickert et al. 2000; Liu et al. 2002; Piedra et al. 2003; Kim et al. 2011; Song et al. 1996; Li et al. 1996). Acute lung injury, including COVID-19, induces substantial ATP release, which activates P2X7R (Cicko et al. 2018; Burnstock 2013; Hasan et al. 2022; Pacheco and Faria 2021). Phosphorylations of *β*-Catenin or p120 catenin promote their dissociation from AJ and activate downstream signaling pathways, including Wnt and Rho GTPase signaling (Huber et al. 1997; Choi et al. 2013). Further investigations on these interactions are necessary to fully elucidate the underlying mechanisms to find possible therapeutic approaches to prevent fibrotic remodeling after early injury such as COVID-19 or other fibrotic diseases.

## Supplementary Information

Below is the link to the electronic supplementary material.

Supplementary file1 (DOCX 28 KB)

## Data Availability

The original data presented in the study are openly available in OPARA at https://opara.zih.tu-dresden.de/handle/123456789/2132.

## Abbreviations

AEC: Alveolar epithelial cell
AJ: Adherens junctions
BLM: Bleomycin
cav-1: Caveolin-1
IF: Immunofluorescence
IHC: Immunohistochemistry
ILD: Interstitial lung disease
PCLS: Precision-cut lung slices
PF: Pulmonary fibrosis
TJ: Tight junctions
WB: Western blot
