# Supplementary material for: The role of alveolar epithelial junctions in early lung injury and COVID-19-induced fibrosis
Source: Histochem Cell Biol. 2026 Sep 11;164(1):85. doi: 10.1007/s00418-026-02533-2 (PMC13569543; doi:10.1007/s00418-026-02533-2)
Supplement: Supplementary file 1 [file 418_2026_2533_MOESM1_ESM.docx]

**THE ROLE OF ALVEOLAR EPITHELIAL JUNCTIONS IN EARLY LUNG INJURY AND COVID-19-INDUCED FIBROSIS**

**Histochemistry and Cell Biology**

**Julián Wiegner^1^, Michael Kasper^1^, Mirko HH Schmidt^1^, Kathrin Barth^1^**

^1^Institute of Anatomy, Medical Faculty “Carl Gustav Carus”, Technical University of Dresden, Fiedlerstr. 42, 01307 Dresden, Germany

**Corresponding author**

Kathrin Barth, Institute of Anatomy, Medical Faculty “Carl Gustav Carus”, Technical University of Dresden, Fiedlerstr. 42, 01307 Dresden, Germany

Tel: +49 351 458 6076

Fax: +49 351 458 6303

E-mail: kathrin.barth@tu-dresden.de

**Supplementary Material**

**Human tissue samples**

Human lung tissue samples were provided as paraffin blocks by the Tumor and Normal Tissue Bank (TNTB) of the NCT/UCC/UKD at the Institute of Pathology of the University Hospital Carl Gustav Carus Dresden, Technical University of Dresden (Fetscherstraße 74, 01307 Dresden, Germany) and were used following approval by the ethics committee of the Technical University of Dresden (EK 59032007; 06 March 2007 and BO-EK-175052020; 22 March 2020). Specimens taken from 22 autopsies were assigned to four groups primarily based on previously described histological features of the diseases, using HE-stained sections from formalin-fixed, paraffin-embedded (FFPE) samples, ensuring comparable ages at death and post-mortem fixation times: acute stage COVID-19 (n = 6, age 57-93 y (average 75 y), post-mortem fixation time average 5.8 d), chronic stage COVID-19 (n = 4, age 59-84 y (average 70.3 y), post-mortem fixation time average 4 d), interstitial lung disease (ILD) of different origin (n = 6, age 65-85 y (average 76.5 y), post-mortem fixation time average 7.2 d), and healthy control lung tissue (n = 6, age 30-87 y (average 67.2 y), post-mortem fixation time average 6.2 d). All tissue samples were classified based on the American Thoracic Society/European Respiratory Society International Multidisciplinary Consensus Classification of the Idiopathic Interstitial Pneumonias [59] and the histological findings in COVID-19 pulmonary tissue [7,8,17]. HE-stained samples in the groups “chronic COVID-19” and “ILD” show characteristic fibrotic changes (myofibroblast proliferation, excessive ECM production, AEC hyperplasia, immune cell infiltration). In the control, ILD and chronic COVID-19 group, negative PCR for SARS-CoV-2 at autopsy or obtainment before the COVID-19 pandemic were required to exclude an acute (re)infection. The FFPE tissues were used for immunohistochemical studies or further processed for mRNA quantification.

**Animal tissue culture**

To further investigate the findings obtained in human tissue, wildtype C57BL/6 mice were purchased from Charles River Laboratories Inc. (RRID: IMSR_JAX:000664, 251 Ballardvale St, Wilmington (MA), USA). The mice housed in the animal facility of the Medical Faculty Carl Gustav Carus, Technical University of Dresden (Fiedlerstr. 42, 01307 Dresden, Germany) with ad libitum access to food and water. The experimental procedures were approved by the state directorate Dresden (DD24-5131/365/19 and TVT17/2016; 20 December 2016). Housing and experimental procedures were conducted in accordance with the Technical University of Dresden Animal Care and Use Committee Guidelines. Mouse lung samples were isolated from female WT mice (n = 4, aged 7 to 9 weeks, weighing 19 to 21 g), sliced for culturing and treating with BLM and processed for immunohistochemistry (IHC) and immunofluorescence (IF) as described in the further methods below, without any exclusions. For sample size determination no formal a priori calculation was conducted. Sample size was chosen based on previous experimental models (including own previous research) using and anticipated effect sizes to detect relevant biological differences; for detection of smaller effects, the study may be underpowered.

**Cell culture**

To further investigate the findings obtained from the retrospective human specimens, we performed in vitro experiments utilizing the immortalized cell line NCI-H441 from ATCC® (RRID: CVCL_1561, Historic District, 10801 University Blvd, Manassas (VA), USA) of human lung origin [60-62].

The cells were cultivated in RPMI-1640 medium (ATCC Corp., Historic District, 10801 University Blvd, Manassas (VA), USA), supplemented with 10 % (v/v) fetal calf serum (FCS, Sigma-Aldrich Chemie GmbH, Riedstraße 2, 89555 Steinheim am Albuch, Germany) at 37 °C with 5 % CO_2_ atmosphere. For the BLM and TGF-β studies, culture medium was supplemented with 1:1.000 insulin, transferrin and selenous acid (ITS) solution (Corning Incorporated, 1 Riverfront Plaza, Corning (NY), USA) and 200 nM dexamethasone (Sigma-Aldrich Chemie GmbH, Riedstraße 2, 89555 Steinheim am Albuch, Germany) to ensure stable experimental conditions [62,63]. NCI-H441 cells were seeded at a density of 2 x 10^5^ cells/mL and passaged twice weekly until passage 75 at a confluency of approximately 60 - 80 %.

For exposure experiments, 100 mU/ml BLM (BLEO-cell®, STADApharm GmbH, Stadastraße 2-18, 61118 Bad Vilbel, Germany) or 1 ng/mL TGF-β (R&D Systems, Inc., 614 McKinley Pl NE, Minneapolis (MN), USA) were added to the culture medium.

For immunohistochemical experiments, NCI-H441 cells were seeded at a density of 1 x 10^5^ cells/mL on cover slips (Engelbrecht Medizin- und Labortechnik GmbH, Tiefenbachweg 13, 34295 Edermünde-Besse, Germany) in well plates and treated with BLM and TGF-β as described above. The cover slips were previously coated with poly-L-lysine using a slightly altered method described by Weinhold [64]. Prior to cell seeding, the cover slips were incubated in a 1:1 ethanol-hydrochloric acid solution (Klinikapotheke des Universitätsklinikums C. G. Carus, Fetscherstraße 74, 01307 Dresden, Germany) for 5 min, 100 % (v/v) ethanol (VWR International GmbH, Le Périgares – Bâtiment B, 201, rue Carnot, 94126 Fontenay-sous-Bois cedex, France), rinsed several times in double distilled wate, followed by incubation in a 0.055 mM solution of poly-L-lysine (Sigma-Aldrich Chemie GmbH, Riedstraße 2, 89555 Steinheim am Albuch, Germany) for 10 min, followed by incubation under UV light. After the treatment of the cells at different time points, the cells were fixed with a 1:1 (v/v) acetone-methanol solution (Merck KGaA, Frankfurter Str. 250, 64293 Darmstadt, Germany; AppliChem GmbH, Ottoweg 4, 64291 Arheilgen, Germany) at -20 °C and subsequently stored at the same temperature.

**Western Blot (WB) analysis**

Whole protein lysates were generated using a lysis buffer containing 20 mM Tris-HCl (Carl Roth GmbH + Co. KG, Schoemperlenstr. 3-5, 76185 Karlsruhe, Germany), pH 7.5, 140 nM NaCl (Carl Roth GmbH + Co. KG, Schoemperlenstr. 3-5, 76185 Karlsruhe, Germany), 1 mM ethylenediamine tetraacetic acid (EDTA, Sigma-Aldrich Chemie GmbH, Riedstraße 2, 89555 Steinheim am Albuch, Germany), pH 8.0, 1 % (v/v) Triton X-100 (ICN Biomedicals, Inc., 1263 South Chillicothe Road, Aurora (OH), USA), 1 tablet protease inhibitor cOmplete Mini, EDTA-free (Roche Diagnostics GmbH, Sandhofer Str. 116, 68305 Mannheim, Germany) and 1 tablet phosphatase inhibitor PhosSTOP (Roche Diagnostics GmbH, Sandhofer Str. 116, 68305 Mannheim, Germany). Protein concentration was determined using the PierceTM bicinchoninic acid (BCA) Protein Assay Kit (23225, Pierce Biotechnology, 3747 N Meridian Rd, Rockford (IL), USA) according to the manufacturer’s instructions. 20 µg of whole protein per sample was transferred into 4x LI-COR® loading buffer (125 mM Tris-HCl, pH 6.8, 50 % (v/v) glycerol (Sigma-Aldrich Chemie GmbH, Riedstraße 2, 89555 Steinheim am Albuch, Germany), 4 % (w/v) sodium dodecyl sulfate (SDS, AppliChem GmbH, Ottoweg 4, 64291 Arheilgen, Germany), 0.2 % (w/v) Orange G (Fluka Chemie AG, Industriestrasse 25, 9470 Buchs, Switzerland), 10 % (v/v) 2-mercaptoethanol). For denaturation, the sample solutions were heated to 95 °C for 5 minutes, loaded on a 10 % (v/v) SDS-polyacrylamide gel at a constant tension of 200 V and transferred by wet blotting at a constant current of 400 mA for 2 hours with a transfer buffer containing 18.2 mM Tris-HCl, 140 mM glycine (SERVA Electrophoresis GmbH, Carl-Benz-Straße 7, 69115 Heidelberg, Germany), 15 % (v/v) methanol and 0.02 % (w/v) SDS. The proteins were transferred on an Immobilon®-FL polyvinylidene difluoride (PVDF) membrane with a pore size of 0.45 µm (Merck Millipore Ltd., Carrigtwohill Tullagreen County Cork, T45 KD29, Ireland).

Subsequently, the total protein on the membrane was stained with the Revert™ 700 Total Protein Stain Kit (926-11010, LI-COR Biosciences, 4647 Superior St, Lincoln (NE), USA) and detected as fluorescent signal on the Odyssey® Fc Imager (RRID: SCR_023227, LI-COR Biosciences, 4647 Superior St, Lincoln (NE), USA), which was used for lane normalization of p120 catenin pan protein. After destaining according to the manufacturer’s requirements, the membrane was blocked with Intercept® (TBS) Blocking Buffer (LI-COR Biosciences, 4647 Superior St, Lincoln (NE), USA) overnight at 4 °C. Incubation with the primary antibody (Table 1) dissolved in Intercept® (TBS) Blocking Buffer (LI-COR Biosciences, 4647 Superior St, Lincoln (NE), USA) was performed for one hour, followed by washing steps and secondary antibody incubation, using the following antibodies: IRDye® 680 goat anti-mouse IgG (RRID: AB_10956588) and IRDye® 800 goat anti-rabbit IgG (RRID: AB_621843, LI-COR Biosciences, 4647 Superior St, Lincoln (NE), USA). Utilizing two different fluorescently labelled secondary antibodies, p120 catenin pan protein and the pY228 form were quantified on the same membrane. The phosphorylated form was normalized to the pan protein. Semi-quantitative analysis was performed with Image StudioTM Lite, Version 5.2.5 (RRID: SCR_013715, LI-COR Biosciences, 4647 Superior St, Lincoln (NE), USA) and statistical comparisons were only carried out on the same blot.

**mRNA quantification using Real-time Reverse Transcription PCR (Real-time RT-PCR)**

For extraction of mRNA, four 5 µm thick FFPE slices of human lung were used. The NucleoSpin® totalRNA FFPE XS Kit (740969.50, Macherey-Nagel GmbH & Co. KG, Valencienner Str. 11, 52355 Düren, Germany) was applied for total RNA extraction, protein and DNA lysis according to the manufacturer’s instructions. RNA concentration was subsequently measured with NanoDrop™ One Microvolume -UV/VIS-Spectrophotometer (RRID: SCR_023005, Thermo Fisher Scientific Inc., 168 3rd Ave, Waltham (MA), USA). For cDNA synthesis, RevertAid H Minus 1st Strand Synthesis Kit (K1622, Thermo Fisher Scientific Inc., 168 3rd Ave, Waltham (MA), USA) was utilized. For each experimental setup, 11 µl of isolated RNA solution was reverse-transcribed employing oligo (dT)18 primers and in another setup using random hexamer primers, which was carried out on the Techne TC-512 Thermal Cycler (Keison Products, 32 Writtle Rd, Chelmsford, UK). Following this, the products were mixed 1:1 and used for PCR. Therefore, SsoAdvanced™ Universal Inhibitor-Tolerant SYBR® Green Supermix (1725270, Bio-Rad Laboratories GmbH, Kapellenstraße 12, 85622 Feldkirchen-Heimstetten, Germany) was used for qPCR on the CFX96 Touch Real-Time PCR Detection System (RRID: SCR_018064, Bio-Rad Laboratories GmbH, Kapellenstraße 12, 85622 Feldkirchen-Heimstetten, Germany), applying the following cycling conditions: initial denaturation 3 min at 98 °C, followed by 40 cycles of amplification (denaturation at 98 °C for 10 sec, primer annealing at 58 °C for 30 sec, elongation at 72 °C for 10 sec) and a final extension at 72 °C for 2 min. For identification of the qPCR products, a melting curve analysis (4 second steps, 0.5 °C each, from 60 °C to 95 °C) followed. The used primers are listed in Table 2. The relative quantification of the mRNA expression was calculated with the ∆∆Ct method using β2-Microglobulin (B2M) and TATA box binding protein (TBP) as housekeeping genes.

**Culture and paraffin embedding of precision-cut lung slices (PCLS)**

Precision cut lung slices were generated as described previously by Held et al. [65] with slight alterations. WT mice were anesthetized and euthanized with an overdose of intraperitoneally administered 5 % (w/v) thiopental (Inresa Arzneimittel GmbH, Ob. Hardtstraße 18, 79114 Freiburg im Breisgau, Germany) and 2.000 international units (IU) heparin (Sigma-Aldrich Chemie GmbH, Riedstraße 2, 89555 Steinheim am Albuch, Germany). Because the animals were anesthetized and euthanized immediately, no adverse events occurred, and no humane endpoints were necessary. After exsanguination by extracting the right kidney, the pulmonary circulation was flushed with phosphate-buffered saline (PBS) buffer (Gibco, 5781 Van Allen Way, Carlsbad (CA), USA) and the lungs were perfused with 0.8 % (w/v) agarose, type VII (low gelling temperature, Sigma-Aldrich Chemie GmbH, Riedstraße 2, 89555 Steinheim am Albuch, Germany). After curing, the lungs were isolated, embedded in 3 % (w/v) agarose and sliced in the Krumdieck tissue slicer (Alabama Research and Development, 152 Metal Samples Rd, Munford (AL), USA) into 500 µm thick PCLS using Dulbecco's Modified Eagle Medium F12 (DMEM/F12), HEPES (Gibco, 5781 Van Allen Way, Carlsbad (CA), USA) as described elsewhere [56,66].

Subsequently, the PCLS were randomly assigned to control or treatment with 300 mU/mL BLM in DMEM/F12 medium being supplemented with 15 mM HEPES, 2.5 mM L-glutamine, 0.5 % (v/v) FCS and 1% (v/v) penicillin/streptomycin (Gibco, 5781 Van Allen Way, Carlsbad (CA), USA) with daily medium change.

After incubation for different durations, the murine lung PCLS were fixed overnight in 4 % (v/v) formalin (SAV Liquid Production GmbH, Hochriesstraße 2, 83126 Flintsbach am Inn, Germany), followed by incubation in 0.1 M Sørensen buffer, pH 7.4 (80 mM disodium hydrogen phosphate dihydrate, 20 mM sodium dihydrogen phosphate monohydrate (Merck KGaA, Frankfurter Str. 250, 64293 Darmstadt, Germany)) for 2 x 30 min and dehydration in 70 % (v/v) ethanol overnight at 4 °C, 96 % (v/v) ethanol for 2 x 30 min, 100 % (v/v) ethanol for 2 x 30 min, xylene (VWR International GmbH, 1-3 Rue d'Aurion, 93110 Rosny-sous-Bois, France) for 20 min and paraffin (Leica Biosystems, Ernst-Leitz-Straße 17-37, 35578 Wetzlar, Germany) for 60 min. Finally the sections were embedded using the EG 1150 (Leica Mikrosysteme Vertrieb GmbH, Ernst-Leitz-Straße 17-37, 35578 Wetzlar, Germany).

**Immunohistochemistry (IHC) using immunoperoxidase and Immunofluorescence (IF)**

For immunohistochemical staining of the human lung and the PCLS of the murine lung, 5 µm paraffin slices were cut with the Microtome (Jung RM 2065, Leica Instruments GmbH, Heidelberger Str. 17-19, 69226 Nußloch, Germany), applied on SuperfrostTMPlus Adhesion slides (Gerhard Menzel GmbH, Saarbrückener Str. 248, 38116 Braunschweig-Lehndorf-Watenbüttel, Germany) and dried at 37 °C overnight. Dewaxing and rehydration were performed by incubation in xylene for 3 x 5 min, 100 % (v/v) ethanol for 3 x 2 min, 96 % (v/v) ethanol for 2 x 2 min, 70 % (v/v) ethanol for 2 x 2 min and distilled water for 2 x 2 min.

Immunoperoxidase staining of the PCLS of murine lung was performed as previously described by Ebeling et al. [66] and according to the supplier’s instructions of the VECTASTAIN® Elite® ABC Kit (RRID: AB_2336821, Vector Laboratories, 30 Ingold Rd, Burlingame (CA), USA). After deparaffinization and rehydration, antigen retrieval was performed in a microwave in sodium citrate buffer, pH 6.0 (1.5 mM citric acid monohydrate (Merck KGaA, Frankfurter Str. 250, 64293 Darmstadt, Germany), 8.5 mM tri-sodium citrate dihydrate (VWR International GmbH, Heinrich-Hertz-Straße 1, 64295 Darmstadt-West, Germany)) for 2 x 5 min at 1.000 W until boiling and subsequently heated with 360 W, followed by 15 min cooling down. After rinsing with distilled water and two washing steps with PBS buffer for 2 x 5 min, the slices were treated with 0.3 % (v/v) hydrogen peroxide solution (Merck KGaA, Frankfurter Str. 250, 64293 Darmstadt, Germany) for 30 min at room temperature followed by two washing steps. Next, the slices were blocked with 1.5 % (v/v) normal serum for 20 min at room temperature, followed by incubation with the primary antibody diluted in PBS buffer overnight at 4 °C. Used antibodies are shown in Table 1. Negative controls were prepared by omitting the primary antibody. After rinsing in PBS for 2 x 5 min, the slices were incubated with the secondary biotinylated antibody for 45 min at room temperature, followed by two more washing steps. Following this, the slides were incubated with the VECTASTAIN ABC Reagent at room temperature for 45 min and rinsed again thereafter. Staining was performed by incubating the sections in 3,3’-diaminobenzidine (DAB, Sigma-Aldrich Chemie GmbH, Riedstraße 2, 89555 Steinheim am Albuch, Germany) as substrate for 7 min and washed in distilled water for 5 min. Counterstaining was carried out with Mayer’s hemalum solution (3,3 mM Hematoxylin, 105,4 mM aluminium potassium sulfate dodecahydrate, 302,3 mM chloral hydrate, 4,8 mM Citric acid monohydrate (all from Merck KGaA, Frankfurter Str. 250, 64293 Darmstadt, Germany), 1 mM sodium iodate (Riedel-de Haën AG, K356 45, 30926 Seelze, Germany)) for 20 sec. Thereafter, the slides were washed in tap water for 10 min, dehydrated using 40 % (v/v) ethanol for 2 min, 70 % (v/v) ethanol for 2 min, 96 % (v/v) ethanol for 2 x 2 min, 100 % (v/v) ethanol for 3 x 2 min and xylene for 3 x 2 min, followed by embedding with DePeX (SERVA Electrophoresis GmbH, Carl-Benz-Straße 7, 69115 Heidelberg, Germany). Immunoperoxidase staining experiments were performed with PCLS of four individual animals. Immunoperoxidase stained tissue was imaged using the ZEISS Axio Scope.A1 microscope and a PLAN-APOCHROMAT 40x/0,95 objective, aberration correction of 0,17, with the Axiocam 503 color camera (2560 x 1920 Pixels, 1 px = 0,115 µm) in the software ZEN 2012, version 1.1.2.0 (RRID: SCR_013672, Carl Zeiss Microscopy GmbH, Königsallee 9-21, 37081 Göttingen-Weststadt, Germany).

IF experiments on the murine PCLS were carried out by the same protocol with the following incubation steps: after the demasking and washing steps, primary antibody incubation overnight at 4 °C, secondary antibody incubation for 1 h at 37 °C, lectin staining with Lycopersicon esculentum (Tomato) Lectin (LEL, TL), biotinylated (1:1.600 in PBS, product no.: B-1175-1, Vector Laboratories (Burlingame (CA), USA)) for 1 h at 37 °C and secondary antibody incubation with Texas Red® Avidin D (1:400 in PBS, product no.: A-2006, RRID: AB_2336751, Vector Laboratories, 30 Ingold Rd, Burlingame (CA), USA) for 1 h at 37 °C, according to a protocol previously described by Barth et al. [67]. After final nucleic staining with 1:2.000 (v/v) DAPI Nucleic Acid Stain (Invitrogen, 5781 Van Allen Way, Carlsbad (CA), USA) the slides were embedded with Fluoromount-GTM (Life Technologies Corp., 5791 Van Allen Way, Carlsbad (CA), USA).

For immunofluorescence staining of the human NCI-H441 cells, cover slips were defrosted and washed with PBS buffer, followed by blocking with 1 % (w/v) BSA (SERVA Electrophoresis GmbH, Carl-Benz-Straße 7, 69115 Heidelberg, Germany) for 30 min at room temperature. The following steps of antibody incubation, dilutions, washing steps, lectin IHC and final embedding were performed as described for murine PCLS, unless antibodies were diluted in 1 % (w/v) BSA.

The human lung slices were bleached in 6 % (v/v) H2O2 for 10 min after rehydrating and, thereafter, demasked in the microwave using EDTA buffer, pH 9.0 (1 mM EDTA disodium salt dihydrate (AppliChem GmbH, Ottoweg 4, 64291 Arheilgen, Germany), 7.7 mM Tris HCl). All further staining steps were performed as described for murine PCLS, except for using different antibody dilutions, see Table 1, and omitting the lectin staining step.

All experimental conditions for each setup were performed equally, including imaging on the same day on the Leica DM6 B fluorescence microscope (RRID: SCR_024857) and a Leica HC PL APO 20x/0,8 objective with the Leica DFC9000 GT camera (2.048 x 2.048 Pixels, 16-Bit detector, space resolution 1 px = 0,162 µm (murine); 1 px = 0,65 µm (human)) in the LAS X software, Version 3.7.6.25997 (RRID: SCR_013673, Leica Microsystems GmbH, Ernst-Leitz-Straße 17-37, 35578 Wetzlar, Germany). All acquisition parameters were kept constant: DAPI was detected with 10% laser intensity, 30 ms acquisition time and a filter set of 350 - 405 nm excitation and 450 - 470 nm emission; Texas Red was detected with 30% laser intensity, 200 ms acquisition time and a filter set of 640 nm excitation and 670 nm emission; FITC was detected with 30% laser intensity, 300 ms acquisition time and a filter set of 470 - 490 nm excitation and 510 - 530 nm emission.

Instant computational clearing mode was used for reducing background noise. For analysing the human specimen and the NCI-H441 cover slips 16 high-resolution fields of view of each sample were merged with LAS X software, the murine lung slices were scanned completely using high-resolution fields and followed by automated image stitching. Analysis was performed in (Fiji is Just) ImageJ, version 2.14.0/1.54f (RRID: SCR_002285, Rasband, W.S., U. S. National Institutes of Health, 9000 Rockville Pike, Bethesda (MD), USA).

For comparison of signal intensity, mean fluorescence intensity (MFI) was measured using a protocol previously described by Shihan et al. [68]. In the human NCI-H441 cells and murine PCLS, the amount of protein of interest inside the cell membrane excluding nuclear signal was investigated. Therefore, the appropriate region of interest was defined by subtracting the area of nuclear signal (DAPI) from the signal of glycocalyx (Lycopersicon esculentum (Tomato) Lectin). In the resulting region of interest, the MFI was calculated and compared between the different samples.

For the human lung samples, a third fluorescent channel was acquired for detecting autofluorescent areas. The area of this channel was subtracted from the signal area of the target protein. MFI was then calculated inside this resulting region of interest. Finally, MFI of negative controls were subtracted.

**Statistical Analysis**

Potential confounding factors were minimized by randomizing the order of treatments and measurements throughout the experimental procedures.

For statistical analysis, at least three independent experimental setups in each approach were performed unless otherwise stated. For experimental approaches of paired samples, the control value was set to 1. Each data set was checked for normal distribution using Shapiro-Wilk test. For significance testing of normally distributed data, a two-tailed paired Student’s t-test for two groups or one-way ANOVA for more groups was performed, followed by a Bonferroni post-hoc hypothesis testing, presented in charts as single data points and means ± standard deviation (SD). If not normally distributed, a Wilcoxon signed-rank test for two groups or ANOVA (Friedman test for paired groups and Kruskal-Wallis test for unpaired groups, followed by Dunn’s post-hoc hypothesis testing) for more than two groups was used, presented in charts as single data points and medians ± interquartile range (IQR) or box-and-whisker plot for better visualisation. The percentages in the text are calculated to the corresponding control value. Statistical analyses were performed using GraphPad Prism, Version 5.03 (RRID: SCR_002798, 225 Franklin Street. Fl. 26. Boston (MA), USA). Significance levels were defined as * p ≤ 0.05 and ** p ≤ 0.01.
